# Anti-Persisters Activity of *Lacticaseibacillus rhamnosus* Culture Filtrates against *Pseudomonas aeruginosa* in Artificial Sputum Medium

**DOI:** 10.3390/ijms25137113

**Published:** 2024-06-28

**Authors:** Marta Bianchi, Semih Esin, Esingül Kaya, Giovanna Batoni, Giuseppantonio Maisetta

**Affiliations:** Department of Translational Research and New Technologies in Medicine and Surgery, University of Pisa, Via S. Zeno 37, 56123 Pisa, Italy; marta.bianchi@phd.unipi.it (M.B.); semih.esin@unipi.it (S.E.); e.kaya@studenti.unipi.it (E.K.)

**Keywords:** persisters, *P. aeruginosa*, antibiotics, biofilm, cystic fibrosis, lactobacilli

## Abstract

Persisters are antibiotic-tolerant bacteria, playing a role in the recalcitrance and relapse of many bacterial infections, including *P. aeruginosa* pulmonary infections in Cystic Fibrosis (CF) patients. Among novel antimicrobial strategies, the use of probiotics and their products is emerging as a particularly promising approach. The aim of this study was to evaluate the anti-persisters activity of culture filtrate supernatants of *Lacticaseibacillus rhamnosus* (LRM-CFS) against *P. aeruginosa* in artificial sputum medium (ASM), which resembles the CF lung environment. Planktonic persisters of two clinical strains of *P. aeruginosa* (PaCF1 and PaCF4) were obtained following two different procedures: (i) exposing stationary-phase cultures to cyanide m-chlorophenylhydrazone (CCCP) in LB medium; (ii) incubating stationary-phase cultures with high doses of tobramycin (128-fold MIC) in ASM. In addition, persisters from biofilm were obtained by exposing 48 h old biofilm of *P. aeruginosa* to 128 x MIC of ciprofloxacin. LRM-CFS at dilutions of 1:6 and 1:4 resulted in being bactericidal in ASM against both PaCF1 and PaCF4 persisters obtained after CCCP or tobramycin treatment. Moreover, LRM-CFS at dilution 1:4 caused a reduction of antibiotic-tolerant bacteria in the biofilm of both *P. aeruginosa* strains. Overall, LRM-CFS represents a promising adjuvant therapeutic strategy against *P. aeruginosa* recalcitrant infections in CF patients.

## 1. Introduction

Cystic Fibrosis (CF) is an autosomic recessive disease due to mutations in the cystic fibrosis transmembrane conductance regulator gene (CFTR) characterized by impaired chloride ion channel function, in which respiratory disease plays a prominent role in morbidity and mortality [1]. The lack of CFTR-mediated chloride secretion leads to the production of hyper-viscous mucus, impaired mucociliary clearance, and bacterial biofilm formation, which cause the failure of immune response within the lung [1]. Although the triple CFTR-modulator therapy has profoundly changed the CF scenario, significantly improving clinical parameters and the life quality of CF patients, the extent to which these therapies may reduce lung infections and restore a healthy-associated lung microbiome has yet to be fully determined [2]. Over the last two decades, inhaled antibiotics (tobramycin, colistin, and aztreonam) have become the preferred therapeutic option, and studies have demonstrated improved pulmonary function and reduced exacerbation rates for inhaled versus oral antibiotics [3]. Despite the improvement of lung functions, current antibiotic treatments fail to eradicate *P. aeruginosa* infection [3]. In addition to genetic resistance mechanisms, the difficulty of eradicating *P. aeruginosa* infections is due to the acquisition of a mucoid phenotype by the infecting strains, the formation of biofilm, and the emergence of a subset of antibiotic-tolerant bacteria known as “persisters” [1,4,5]. Biofilm-residing bacteria exhibit significantly elevated antibiotic resistance, up to 1000 times more, than planktonic bacteria. The mechanisms for the antibiotic resistance of bacteria in biofilms are multiple. The first resistance mechanism is represented by the barrier effect played by the extracellular matrix of the biofilm, composed of exopolysaccharide, DNA, and proteins [6,7]. Secondly, if an antibiotic can move through the superficial layers of the biofilm, it encounters a microenvironment rich in catabolites and poor in oxygen that could impact antibiotic activity [6,7]. A third mechanism that contributes to the antimicrobial resistance of biofilm is the presence of persister cells in the deep biofilm layers [6,7].

Persister cells are a subset of dormant cells, which lack proton motive force, transcription, and translation [8]. Although persister cells can arise stochastically in growing cultures, their formation can also be induced after exposure to antibiotic treatment, nutrient and oxygen deprivation, and oxidative stress [8]. The identification of new antimicrobial approaches able to act also against persister cells is needed. In this context, the possible use of probiotics to control respiratory infections in CF patients represents a recently emerged and particularly attractive strategy [9,10,11,12]. To replace the use of live probiotics, thus taking into account safety concerns, different strategies have been developed, including the use of killed probiotics, microbial extracts, and cell-free supernatants [13,14,15]. Overall, these inanimate preparations of probiotics have been referred to as “postbiotics”, but a debate still exists on the proper use of this term [16]. In postbiotics prepared from lactobacilli, several bioactive metabolites such as organic acids, short-chain fatty acids, and antimicrobial peptides have been described as [17]. Recently, we demonstrated a strong and fast antibacterial activity of cell-free supernatants from *Lacticaseibacillus rhamnosus* (LRM-CFS) in artificial sputum medium (ASM) towards clinical strains of *P. aeruginosa* from CF lung, both in the planktonic and biofilm mode of growth, and a marked activity of the same supernatants against biofilms of wound pathogens in in vivo-like conditions [18,19].

In this study, we tested in ASM the ability of LRM-CFS to kill persister cells of *P. aeruginosa* generated either by exposing bacterial cells to cyanide m-chlorophenylhydrazone (CCCP) [20], an unspecific cytoplasmic membrane uncoupler, or to high concentrations of tobramycin. Moreover, LRM-CFS were tested on persisters of *P. aeruginosa* obtained from biofilm exposed to high doses of ciprofloxacin. Overall, the results obtained demonstrated that, unlike conventional antibiotics, LRM-CFS markedly affected the vitality of persister cells of *P. aeruginosa* in conditions relevant to the CF lung environment.

## 2. Results

### 2.1. Bactericidal Activity of LRM-CFS in ASM against CCCP-Generated P. aeruginosa Persisters

**Figure 1:**
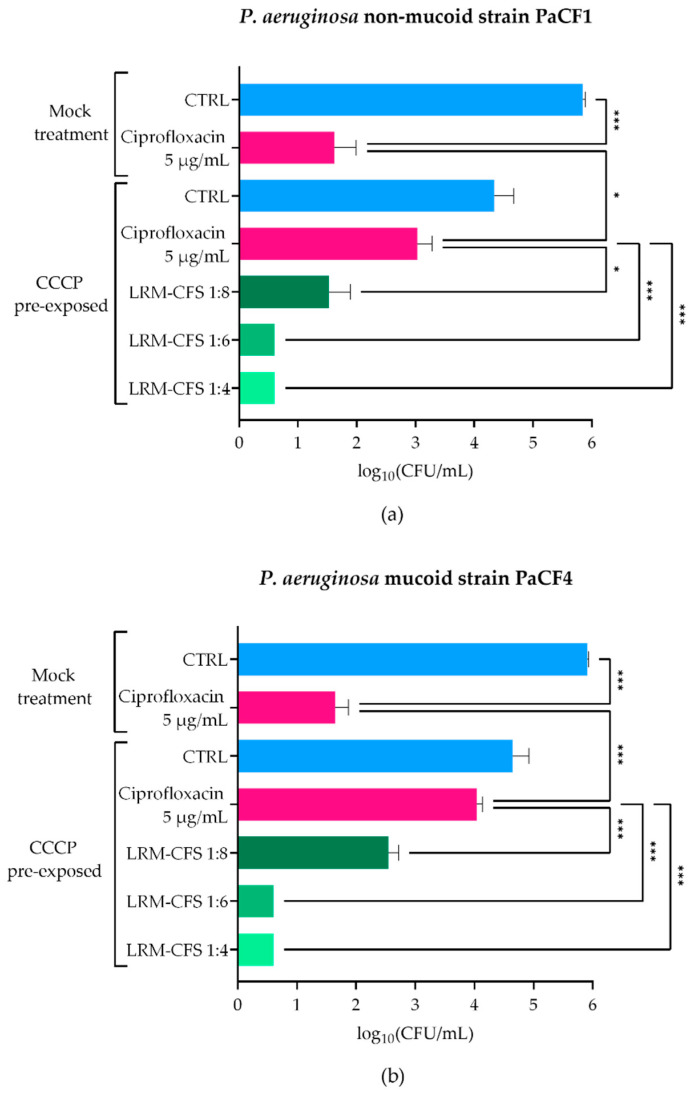
Killing activity of LRM-CFS on CCCP-generated persisters of PaCF1 (**a**) and PaCF4 (**b**) in ASM. PaCF1 and PaCF4 persisters were generated by exposure of stationary-phase cultures to CCCP 200 μg/mL for 3 h in LB. LRM-CFS were diluted at 1:4, 1:6, and 1:8 in de Man, Rogosa, and Sharpe broth (MRSB) and tested against persisters of PaCF1 (**a**) and PaCF4 (**b**) strains in ASM. After 3 h of incubation, samples were serially diluted and plated on solid medium for CFU counts. Samples denominated “mock treatment” represent control bacteria not pre-incubated with CCCP. CTRL: untreated bacteria incubated in ASM with De Man-Rogosa-Sharpe broth (MRSB) pH 4 diluted at 1:4; Ciprofloxacin was tested at 5 μg/mL (20 x MIC for both strains). Results are shown as mean ± standard error of the mean values (*n* = 4). Statistical significance was evaluated by one-way ANOVA followed by the Tukey–Kramer post hoc test. * *p* < 0.05, *** *p* < 0.001.

To explore the LRM-CFS therapeutic potential in conditions closely resembling the CF lung, we assessed the anti-persisters activity of LRM-CFS in ASM, which mimics the nutritional environment of the CF lung. To obtain *P. aeruginosa* persisters, two CF clinical isolates (PaCF1 and PaCF4), exhibiting a non-mucoid and a mucoid phenotype, respectively, were exposed to CCCP for 3 h in LB medium, according to a previously described procedure [20]. The ability of CCCP to induce persistence was confirmed by exposing CCCP-pretreated cultures to ciprofloxacin at very high concentration (5 µg/mL corresponding to 20 x MIC) in ASM. Ciprofloxacin displayed a modest effect against CCCP-pretreated bacteria of both *P. aeruginosa* strains, whereas it showed a marked bactericidal activity against mock-pretreated bacteria (Figure 1a,b), confirming the acquisition of a ciprofloxacin-tolerant status of CCCP-pretreated bacteria. The bactericidal activity of LRM-CFS against CCCP-generated persisters of *P. aeruginosa* was then evaluated after 3 h of incubation in ASM. LRM-CFS used at dilutions of 1:6 and 1:4 eradicated persister-enriched cultures to the limit of detection (5 CFU/mL) for both *P. aeruginosa* strains (PaCF1 and PaCF4) (Figure 1a,b). When tested at the dilution 1:8, LRM-CFS caused a statistically significant decrease in the CFU/mL number of both *P*. *aeruginosa* strains, accounting for approximately 1.5-log compared to the number of persister bacteria surviving to ciprofloxacin treatment (Figure 1a,b).

### 2.2. Bactericidal Activity of LRM-CFS in ASM against Tobramycin-Selected P. aeruginosa Persisters

**Figure 2:**
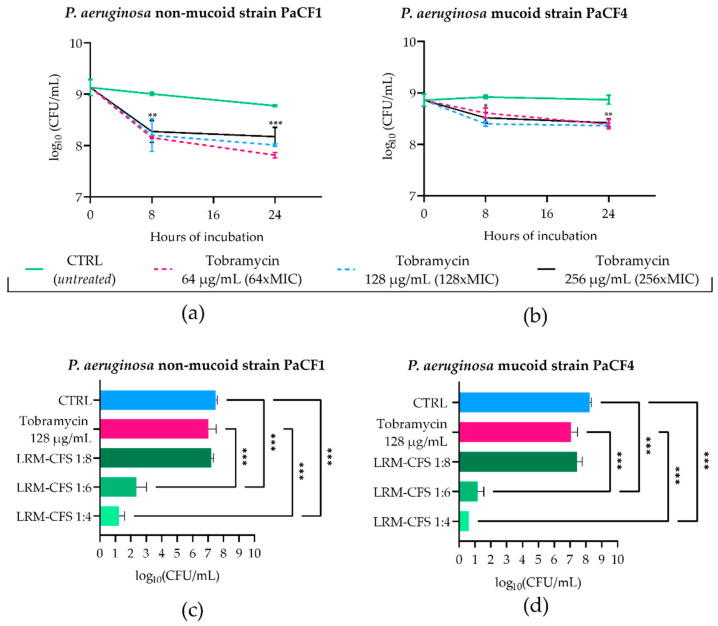
Kinetics of PaCF1 (**a**) and PaCF4 (**b**) persister selection in ASM. Bacteria were exposed to tobramycin at concentrations of 64 μg/mL, 128 μg/mL, and 256 μg/mL, and their viability was assessed at 8 h and 24 h time points by CFU counts. Killing activity of LRM-CFS diluted at ratios of 1:4, 1:6, and 1:8 was evaluated against persisters of PaCF1 (**c**) and PaCF4 (**d**) in ASM after 24 h of incubation. In (**c**,**d**)—CTRL: persisters selected by 24 h tobramycin exposure (as shown in (**a**,**b**)) and incubated in ASM in the presence of MRSB pH 4; tobramycin represents persister bacteria further incubated in ASM with tobramycin 128 μg/mL. Results are shown as mean ± standard error of the mean values (*n* = 4). Statistical significance was evaluated by two-way ANOVA for (**a**,**b**) and one-way ANOVA for (**c**,**d**), followed by the Tukey–Kramer post hoc test. * *p* < 0.05, ** *p* < 0.01, *** *p* < 0.001.

To mimic the in vivo generation of persister cells following antibiotic treatment in CF-relevant conditions, stationary phase cultures of PaCF1 and PaCF4 were exposed to concentrations well above the MIC values of tobramycin (MIC: 1 μg/mL) in ASM. To this aim, the two strains were incubated with tobramycin at 64, 128, and 256 μg/mL, and the surviving bacterial load was evaluated after 8 and 24 h. As shown in Figure 2a,b, after 8 h of incubation, tobramycin at all three concentrations tested caused a slight decrease in the CFU number of both *P. aeruginosa* strains. Such numbers remained almost constant at 24 h irrespective of the antibiotic dose used. The characteristic biphasic killing curves obtained, reaching a plateau at increasing exposure times, were indicative of the presence of persister cells. To verify that the observed tolerance of persister cells to tobramycin was not due to the acquisition of resistance mechanisms, MIC values of tobramycin towards both *P. aeruginosa* strains were determined before persistence induction and after removal of the antibiotic and resuspension of the persistent population in fresh medium. For both bacterial strains, MICs of tobramycin either before or after persister reactivation were 1 μg/mL, confirming that the tobramycin tolerance of *P. aeruginosa* strains was transient and not due to resistance mechanisms.

To test the antimicrobial effect of LRM-CFS on tobramycin-tolerant *P. aeruginosa*, bacteria pre-exposed to 128 μg/mL of tobramycin for 24 h in ASM were further exposed for 24 h to LRM-CFS (diluted at 1:8, 1:6, and 1:4) or to tobramycin 128 μg/mL in fresh ASM. The choice of 24 h as the exposure time of *P. aeruginosa* to LRM-CFS in ASM was due to the inefficacy of supernatants at earlier times (data not shown). Both *P. aeruginosa* strains again displayed high levels of tolerance to tobramycin tested at 128 μg/mL (Figure 2c,d). Similar results were obtained with LRM-CFS diluted at 1:8. In contrast, LRM-CFS diluted at 1:6 caused a decrease in the viable bacteria number of approximately 5-log and 6-log against PaCF1 and PaCF4, respectively, compared to that of bacteria exposed to tobramycin (Figure 2c,d). LRM-CFS diluted at 1:4 killed almost all bacteria, with the number of survival cells under (PaCF4) or close (PaCF1) to the limit of detection (5 CFU/mL) (Figure 2c,d). To verify the antibacterial activity of tobramycin in ASM, PaCF1 and PaCF4 strains in the exponential growth phase were incubated in ASM with tobramycin 128 μg/mL for 24 h. We observed a marked bactericidal effect of tobramycin against both bacterial strains, confirming that the lack of bactericidal activity of tobramycin against persisters was due to their tolerant state (Supplementary Figure S1).

### 2.3. Bactericidal Activity of LRM-CFS against P. aeruginosa Persisters from Biofilm

**Figure 3:**
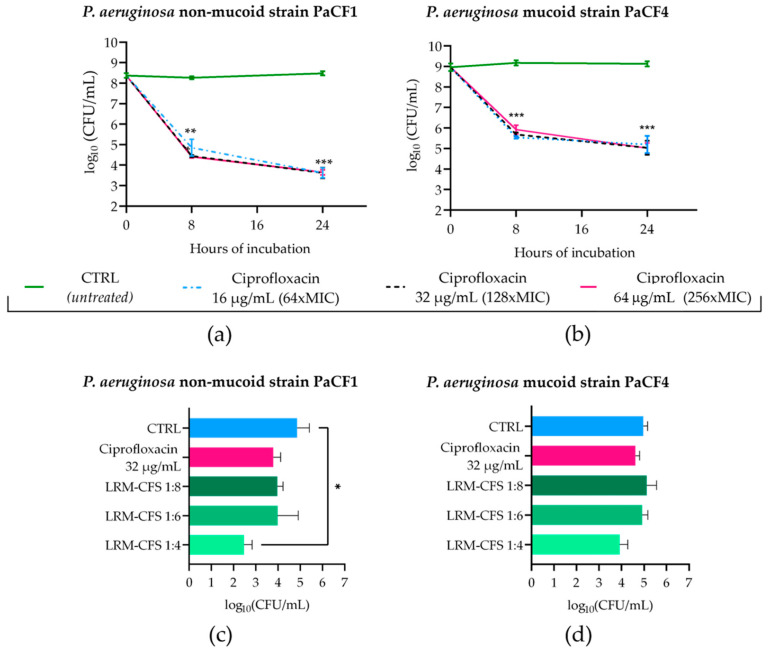
Kinetics of PaCF1 (**a**) and PaCF4 (**b**) persister selection from biofilm in ASM. Bacteria were exposed to ciprofloxacin at concentrations of 16 μg/mL, 32 μg/mL, and 64 μg/mL, and their viability was assessed at 8 h and 24 h time points by CFU counts. Killing activity of LRM-CFS diluted at ratios of 1:4, 1:6, and 1:8 was evaluated against persisters of PaCF1 (**c**) and PaCF4 (**d**) after 24 h of incubation in ASM. In (**c**,**d**)—CTRL: persisters selected with ciprofloxacin 32 μg/mL from biofilm and incubated with MRSB pH 4; ciprofloxacin represents persisters selected with ciprofloxacin at 32 μg/mL and further incubated with ciprofloxacin 32 μg/mL. Results are shown as mean ± standard error of the mean values (*n* = 4). Statistical significance was evaluated by two-way ANOVA for (**a**,**b**) and one-way ANOVA for (**c**,**d**), followed by the Tukey–Kramer post hoc test. * *p* < 0.05, ** *p* < 0.01, *** *p* < 0.001.

To obtain *P. aeruginosa* persisters from biofilm in CF-relevant conditions, 48 h old biofilms of PaCF1 and PaCF4 grown in ASM were exposed to concentrations of ciprofloxacin much higher than the MIC value (MIC: 0.25 μg/mL). To this aim, mature biofilms of both strains were incubated with ciprofloxacin at 16, 32, and 64 μg/mL in ASM, and the biofilm-associated viable bacteria count was evaluated after 8 and 24 h of incubation. As shown in Figure 3a,b, ciprofloxacin at all three concentrations tested caused a significant decrease in the CFU number of both *P. aeruginosa* strains after 8 h of incubation, but the number of surviving bacteria remained almost stable at 24 h. The biphasic killing kinetic was suggestive of the successful selection of ciprofloxacin-tolerant bacteria. The activity of LRM-CFS diluted at 1:4, 1:6, and 1:8 was then tested on bacteria that had been previously exposed to ciprofloxacin at 32 μg/mL (128 x MIC) for 24 h in ASM.

When tested at the dilution of 1:4, LRM-CFS caused approximately 2-log and 1-log reductions in the CFU number of PaCF1 and PaCF4, respectively, compared to the control. At the same concentration, around 1-log reduction in the CFU number of both *P. aeruginosa* strains was observed, as compared to ciprofloxacin-treated samples. LRM-CFS at the dilutions of 1:6 and 1:8 caused a decrease of 1-log in the PaCF1 CFU number compared to the control (Figure 3c), whereas they resulted in being inactive against the PaCF4 strain.

### 2.4. Cytotoxicity

**Figure 4:**
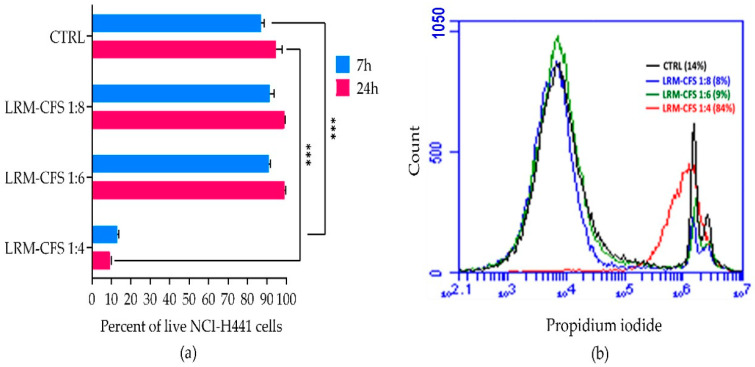
Cytotoxic effect of the LRM-CFS diluted at 1:4, 1:6, and 1:8 in complete RPMI medium was tested in vitro against NCI-H441 cell line grown on the surface of collagen scaffolds in the 3D lung epithelium model. Cells were stained for 5 min with 0.1 μg/mL propidium iodide at room temperature before performing flow cytometric acquisition. (**a**) Percentage of live NCI-H441 cells after exposure to LRM-CFS for 7 and 24 h. (**b**) Overlay histogram data from a representative experiment showing PI-positive NCI-H441 percentages (dead cells). CTRL: MRSB diluted at 1:4 in complete RPMI medium. Results are shown as mean ± standard error of the mean values (*n* = 3). Statistical significance was evaluated by one-Way ANOVA followed by the Tukey–Kramer post hoc test. *** *p* < 0.001.

The cytotoxic activity of LRM-CFS diluted at 1:4, 1:6, and 1:8 in complete RPMI medium was tested in vitro against the human distal lung epithelium cell line NCI-H441, grown on the surface of collagen scaffolds to mimic the architecture of the lung tissue [21] in order to evaluate its translational potential. Following a 7 h incubation, a cytotoxic effect of less than 10 percent was observed for 1:6- and 1:8-diluted LRM-CFS, while when LRM-CFS was diluted at 1:4, the cytotoxicity was around 85% (Figure 4a,b). Cytotoxic activity of LRM-CFS remained low even after a longer (24 h) incubation (Figure 4). MRSB diluted at 1:4 with complete RPMI (CTRL) showed cytotoxicity around 10 percent.

## 3. Discussion

Generation of persisters in vivo allows subpopulations of bacteria to survive under antibiotic treatment and may account for the recalcitrance of most chronic infections and antibiotic treatment failure [22]. Persisters formation is a complex survival strategy and is correlated with various factors that include growth phase, expression of stringent response genes, SOS response, quorum sensing and toxin–antitoxin systems [23]. In this study, we aimed to test the ability of LRM-CFS to kill persister cells of *P. aeruginosa* both in planktonic and biofilm models of growth in ASM, resembling the CF lung environment.

Persister-enriched cultures were first obtained by chemical treatment of stationary phase cultures with CCCP, a membrane uncoupling agent [20]. We have previously demonstrated that CCCP-treated cultures undergo a general reduction of metabolic activity, as assessed by monitoring bacterial heat production through isothermal microcalorimetry and by evaluating oxidoreductase activity by flow cytometry [20]. Furthermore, we have reported that after CCCP removal, induced persisters show a lag phase before the resumption of normal growth and a reversion to an antibiotic-sensitive phenotype, following metabolic reactivation [20]. All these observations are compatible with the acquisition of a CCCP-induced persister-like status that has been linked to the inhibition of ATP synthesis and the consequent reduction in bacterial metabolic activity [24]. Interestingly, by employing the same procedure in the present study, we demonstrated the ability of LMR-CFS to exert a bactericidal effect against CCCP-generated persister cells of *P. aeruginosa* in ASM. In contrast, ciprofloxacin, an antibiotic commonly used for the therapy of CF lung infections, was inactive when tested at very high concentration (20 x MIC). Several activities have been ascribed to supernatants from lactic acid bacteria, including antibacterial, antibiofilm, antioxidants, antivirulence, and wound-healing activity [18,25,26], but to the best of our knowledge, anti-persister activity has never been reported before.

In the attempt to mimic the in vivo generation of persister cells in the lungs of CF patients, in this study we further explored the anti-persister activity of LMR-CFS against persister-enriched populations of *P. aeruginosa* obtained through exposure to high antibiotic concentrations in ASM. It has been demonstrated that upon antibiotic exposure, the stringent response (*relA* and *spoT*) is induced, leading to an increase in (p)ppGpp, which in turn arrests the cell growth (by directly interacting with RNA polymerase and inhibiting the transcription) providing a fitness advantage in stressful conditions [27]. Thus, in this study, we also selected persisters directly in ASM after exposure of *P. aeruginosa* to high doses (128 x MIC) of tobramycin, an antibiotic commonly used for the therapy of lung infections by *P. aeruginosa* in CF patients. We observed that tobramycin at very high concentrations (up to 256-fold MIC) in ASM caused only a mild decrease in viable bacteria from stationary-phase cultures of both *P. aeruginosa* strains tested, and the killing curves were typically biphasic, suggesting the formation of persisters in the adopted conditions. The acquisition of a tobramycin tolerance state was confirmed by the marked survival of bacteria observed following a second exposure of the cultures at high dose of the same antibiotic. Despite the high number of tobramycin-tolerant bacteria, LRM-CFS (diluted at 1:6 and 1:4) were able to exert a strong bactericidal effect, causing a decrease in the CFU number under or close to the limit of detection. This point is of particular importance, as eradication of the entire bacterial population is mandatory to avoid the relapse of infections and minimize the chance of resistance development during prolonged antibiotic treatments.

The antibacterial activity of CFS from lactobacilli is mainly ascribed due to the acidic pH caused by the presence of weak organic acids (WOAs) (i.e., lactic, acetic, and propionic acids) [28]. The presence of high levels of lactic acid (approximately 0.15 M) was confirmed in our LRM-CFS preparation. Previous studies have suggested several potential mechanisms of antimicrobial action for WOAs [29,30], some of which could be involved in the anti-persisters activity of LRM-CFS observed in the present study. As WOAs are relatively hydrophobic, they can diffuse across the bacterial cell membranes, dissociate, and lower the cytoplasmic pH of the bacteria. Such a decrease in the cytoplasmic pH can also increase the osmolality, resulting in an influx of water and subsequent increase in turgor pressure [31]. Another suggested mechanism of action for WOAs is the permeabilization of the outer membrane of Gram-negative bacteria [32]. The lipophilic nature of some organic acids implies that they can migrate and intercalate in the lipid membranes of the bacterial cell envelope, with potentially toxic consequences [32]. The likely membrane-targeting mechanism of action of WOAs could explain the observed ability of LRM-CFS to kill dormant persister cells more efficiently than conventional antibiotics (e.g., tobramycin) that, instead, act mostly on actively replicating bacterial cells [20]. Nonetheless, we cannot exclude a contribution to the anti-persister activity of LRM-CFS of the bacteriocins. Indeed, the genome sequence of *L. rhamnosus* GG strains (GenBank accession no. FM179322), which was used in this study, indicates the presence of bacteriocin genes biosynthetic proteins (LRHM_2289 to LRHM_2312). Moreover, we produced LRM-CFS in MRS broth at pH 6.2 and 37 °C, which were previously determined as the optimal conditions for bacteriocin production [33]. Of note, WOAs and bacteriocins might synergize against persister bacteria; in fact, WOAs might have a destabilizing effect on the outer membrane of Gram-negative bacteria, favoring bacteriocins reaching their main target represented by the cytoplasmic membrane [32].

The biofilm lifestyle represents a reservoir of high phenotypic diversity, and it is considered one of the most important adaptive mechanisms of *P. aeruginosa* within the CF lung [34,35]; yet, the transition towards the persister phenotype in *P. aeruginosa* is not fully elucidated. Biofilms have a high tolerance towards antibiotics, which makes their eradication extremely challenging, urging the need to find new antibiofilm strategies [36,37].

It has been demonstrated that certain antibiotics, such as gentamicin and ciprofloxacin, can induce the formation of highly tolerant dormant cells in biofilms, explaining, at least partially, the biofilm recalcitrance to antimicrobial treatments [23]. Thus, in this study, we lastly attempted to select *P. aeruginosa* persisters in ASM, exposing biofilms of both PaCF1 and PaCF4 strains to high doses of ciprofloxacin. The biphasic killing curve obtained was indicative of a successful enrichment of ciprofloxacin-tolerant persister cells in the biofilm. Treatment of these cells with LRM-CFS caused an approximately 1-log decrease in the number of ciprofloxacin-tolerant bacteria, but only when the supernatants were used at the highest concentration (1:4) and towards the non-mucoid strain. In a previous study, we observed that LRM-CFS exerted a marked bactericidal effect on mature biofilms of both PaCF1 and PaCF4 strains [18]. Differently, in this study, a relatively poor activity of LRM-CFS against biofilm-associated persisters was observed. This observation, together with the marked bactericidal effect observed instead against planktonic persisters, suggests that tolerant bacteria in biofilms, generated by antibiotic treatment, may display peculiar features that render them particularly refractory to antimicrobials, including LRM-CFS.

Assessment of the cytotoxic effect of LRM-CFS towards a cell line relevant for the lung environment demonstrated good tolerability at concentrations able to target planktonic persisters (1:6, 1:8), while a marked cytotoxic effect was observed at the dilution of 1:4. Although no adverse effect of LRM-CFS was recently reported by us in an in vivo *Galleria mellonella* model [18], future strategies aimed at minimizing the cytotoxic potential of LRM-CFS on host cells are warranted. These may include the development of appropriate CFS carriers/formulations, as recently reported by Sharaf and co-workers [38].

To our knowledge, this study for the first time describes the selection of *P. aeruginosa* persisters in planktonic and biofilm conditions in ASM using an antibiotic. Only two strains of *P. aeruginosa* were tested, and this may represent a limit of our study. Nevertheless, the two strains were representative of the two major *P. aeruginosa* phenotypes (mucoid and non-mucoid) found in CF patients and did not show any major difference in the efficacy of persister formation. Our model may represent a useful method to test new anti-persister agents in CF-relevant conditions. Further studies on anti-persisters activity of LRM-CFS in in vivo models are needed to fully explore their potential to eradicate persistent infections. Nevertheless, the results obtained in this study suggest that LRM-CFS could be potentially administered to CF patients during the antibiotic-free intervals, to decrease the number of *P. aeruginosa* persisters selected by the recurrent antibiotic treatments. Studies are underway in our laboratory to develop a liquid LMR-CFS formulation for aerosol administration, in view of future evaluations of CFS as inhaled therapy to treat or prevent *P. aeruginosa* lung infections [39].

## 4. Materials and Methods

### 4.1. Bacterial Strains and Culture Conditions

The *Pseudomonas aeruginosa* strains used in this study, namely, PaCF1 (non-mucoid) and PaCF4 (mucoid), were obtained from sputum samples of chronically infected cystic fibrosis patients. Identification of such bacterial strains was conducted using a MALDI-TOF MS (Bruker Daltonics, Bremen, Germany) Microflex LT Mass Spectrometer with MALDI Biotyper 3.1 software (Bruker Daltonics, Bremen, Germany), following the manufacturer’s protocol. A score ≥ 2.00 allowed identification at the species level, as previously described [18]. For determining colony forming units (CFUs), *P. aeruginosa* was plated on Tryptone Soy Agar (TSA, Oxoid, Basingstoke, Hampshire, UK). *L. rhamnosus* was isolated from a commercial product available on the market, as described in our previous work [18]; briefly, probiotic capsules were dissolved in water and streaked on the De Man–Rogosa–Sharpe agar (Oxoid, Basingstoke, Hampshire, UK) to isolate single colonies. Colonies were picked up and identified at the species level by MALDI-TOF MS, as described earlier. The *L. rhamnosus* strain was further confirmed as GG through whole genome sequencing and subsequent data analysis by Novogene (Beijing, China) [18]. Bacterial strains were grown in Luria-Bertani broth (*P. aeruginosa*) (LB, Sigma-Aldrich, St. Louis, MO, USA) or in De Man–Rogosa–Sharpe broth (*L. rhamnosus*) (MRSB, Oxoid, Basingstoke, Hampshire, UK). For stock culture preparation, cultures at late-log phase were subdivided in aliquots and kept frozen at −80 °C until use.

### 4.2. L. rhamnosus Culture Filtrate (LRM-CFS) Preparation

*L. rhamnosus* was cultured in MRSB under shaking conditions for 48 h. The cultures obtained were centrifugated at 4000× *g* for 10 min, and the supernatants were filtered with 0.22 µm filters (Euroclone SpA, Pero, Milan, Italy). The resultant LRM-CFS were aliquoted and stored frozen at −20 °C until use.

### 4.3. Preparation of Artificial Sputum Medium (ASM)

For the preparation of ASM, the specified quantities of components were dissolved in 20 mL of sterile water: 100 mg mucin from pig stomach (Sigma-Aldrich); 80 mg unsheared salmon sperm DNA (Sigma-Aldrich); 100 mg NaCl; 44 mg KCl; 0.1 mL egg yolk emulsion (Sigma-Aldrich); 0.12 mg diethylentriaminepentaacetic acid (Sigma-Aldrich); 100 mg casamino acids (Gibco); 11.4 mg glucose (Sigma-Aldrich). The pH of the solution was adjusted to 6.8 with HCl [40]. Each experiment was conducted using either freshly prepared ASM or ASM that had been stored at 4 °C for a maximum of one week.

### 4.4. Reagents

A stock solution of CCCP (Sigma-Merck) was prepared by diluting it in dimethyl sulfoxide (DMSO) to reach a concentration of 10 mg/mL. The resulting stock solution was then aliquoted and stored at −20 °C.

A stock solution of 5 mg/mL of tobramycin and ciprofloxacin (Sigma-Merck) was prepared in sterile water and stored in aliquots at −20 °C.

### 4.5. Determination of Minimum Inhibitory Concentrations (MICs)

The minimum inhibitory concentrations (MICs) of tobramycin against PaCF1 and PaCF4 were determined following the standard broth microdilution method recommended by The European Committee on Antimicrobial Susceptibility Testing (EUCAST http://http://www.eucast.org/clinical_breakpoints accessed on 1 May 2024). Briefly, bacterial cultures were grown in Mueller–Hinton broth (MHB, Oxoid, Basingstoke, Hampshire, UK) until reaching the exponential growth phase, then diluted in the same medium to achieve a final density of 5 × 10^5^ CFU/mL. MIC values were defined as the lowest concentration of tobramycin that resulted in the inhibition of visible growth after 24 h of incubation at 37 °C.

### 4.6. CCCP Induction of P. aeruginosa Planktonic Persisters and Evaluation of LRM-CFS Activity

Overnight cultures of PaCF1 and PaCF4 in Luria Bertani broth (LB, Oxoid, Basingstoke, Hampshire, UK) were incubated with CCCP 200 μg/mL for 3 h at 37 °C with shaking in an Eppendorf ThermoMixer^®^ C at 550 rpm, as previously described [20]. A control sample was prepared by adding the same amount of DMSO, used to dissolve CCCP, and incubating it under the same conditions.

After exposure to CCCP, *P. aeruginosa* persister cells were exposed to LRM-CFS diluted at 1:4, 1:6, and 1:8 for 3 h at 37 °C. At this aim, persister bacteria were washed twice with PBS to remove CCCP by centrifugation at 1700× *g* for 10 min, and then they were resuspended in ASM in the presence of LRM-CFS diluted at 1:4, 1:6, and 1:8. To confirm the persistence condition of bacteria, in each set of experiments, two control samples were prepared: CCCP-pretreated bacteria were incubated with ciprofloxacin 5 μg/mL (20 x MIC) or with MRSB at pH = 4 (at the same volume of LRM-CFS). Samples were incubated for 3 h in shaking conditions at 550 rpm, at the temperature of 37 °C in an Eppendorf ThermoMixer^®^ C (Eppendorf, Hamburg, Germany). After incubation, samples were centrifuged and washed again as described before, serially diluted, and plated on TSA. TSA plates were incubated for 24–72 h for CFU enumeration.

### 4.7. Tobramycin Selection of P. aeruginosa Planktonic Persisters in ASM and Evaluation of LRM-CFS Activity

Overnight cultures of PaCF1 and PaCF4 grown in LB were centrifuged at 1700× *g* for 10 min, and the bacterial pellet was re-suspended in ASM. Tobramycin was added to the ASM at concentrations of 64 μg/mL (64 x MIC), 128 μg/mL (128 x MIC), and 256 μg/mL (256 x MIC). Samples were then incubated at 37 °C in shaking conditions at 550 rpm in a ThermoMixer (Eppendorf). A control condition was prepared by adding water instead of tobramycin and incubated in the same conditions.

At time 0, 8 h, and 24 h of incubation with tobramycin, the samples were centrifuged at 2200× *g* for 10 min, and the bacterial pellet was washed two times with PBS, diluted, and plated on TSA. TSA plates were incubated for 24–72 h for CFU enumeration.

To ensure that the observed phenomenon was not due to the development of resistance, a standard MIC test was performed after the second tobramycin exposure, with the method previously described in Section 4.5.

*P. aeruginosa* persisters obtained after exposure to tobramycin were exposed to LRM-CFS diluted at 1:4, 1:6, and 1:8 for 24 h. Persister bacteria were washed twice with PBS to remove tobramycin by centrifugation at 2200× *g* for 10 min, and then they were resuspended in ASM in the presence of LRM-CFS diluted at 1:4, 1:6, and 1:8. To confirm the maintenance of the persistent status, in each set of experiments, persisters obtained with tobramycin 128 µg/mL were incubated again with tobramycin at the concentration of 128 µg/mL. An additional control was represented by persisters incubated with MRSB at pH = 4 at the same volume of LRM-CFS. Samples were incubated for 24 h in shaking conditions at 550 rpm, at the temperature of 37 °C in an Eppendorf ThermoMixer^®^ C. After incubation, samples were centrifuged and washed again as described before, serially diluted, and plated on TSA. TSA plates were incubated for 24–72 h for CFU enumeration.

### 4.8. Ciprofloxacin Selection of P. aeruginosa Biofilm Persisters in ASM and Evaluation of LRM-CFS Activity

Overnight cultures of PaCF1 and PaCF4 grown in LB were diluted at 1:50 in ASM and seeded at the volume of 100 μL into wells of a polystyrene 96-well microtiter plate. The microtiter plate was incubated in static conditions at 37 °C to let biofilms grow. After 24 h of incubation, the medium was changed with fresh ASM and incubated for an additional period of 24 h, resulting in a total incubation period of 48 h. Following incubation, wells were gently washed thrice with PBS to remove non-biofilm-embedded bacteria and resuspended in ASM with ciprofloxacin concentrations of 16 μg/mL (64 x MIC), 32 μg/mL (128 x MIC), and 64 μg/mL (256 x MIC) and incubated at 37° C for 8 h and 24 h.

Following incubation, wells were washed thrice with PBS to remove non-biofilm-embedded bacteria. A sterile tip was used to detach biofilms from the surface of each well, and biofilm-derived cells were resuspended in 1 mL of PBS. To obtain single-cell suspensions, biofilm cells were vortexed for 30 s, sonicated for 30 s in a water bath sonicator (Ultrasonic cleaner, VWR), and vortexed for a further 30 s. The CFU count of biofilm-associated bacteria was performed at time 0, after 8 h and 24 h incubation with ciprofloxacin. To this aim, bacterial suspensions were serially diluted, plated on TSA agar plates, and incubated for 24–72 h for CFU enumeration.

The persister cells obtained after exposure of biofilm with ciprofloxacin at the concentration of 32 μg/mL were treated with LRM-CFS diluted at 1:4, 1:6, and 1:8 for 24 h. To confirm the antibiotic persistence of *P. aeruginosa* in the treated biofilm, all the experimental samples were simultaneously treated with ciprofloxacin a second time at the same concentration (32 µg/mL). Biofilm persister bacteria were washed thrice with PBS to remove ciprofloxacin and were then resuspended in ASM in the presence of LRM-CFS and incubated in static conditions at the temperature of 37 °C. Following incubation, wells were gently washed thrice with PBS to remove non-biofilm-embedded bacteria. Biofilm-associated bacteria in wells were disaggregated as previously described in this subsection, and bacterial suspensions were serially diluted on TSA agar plates and incubated for 24–72 h for CFU enumeration. A control condition was prepared by adding the same amount of MRSB at pH = 4 and incubating in the same conditions.

### 4.9. Cytotoxicity

The cytotoxic potential of the LRM-CFS was tested against the human lung adenocarcinoma cell line NCI-H441, kindly provided by Professor Anna Maria Piras from the University of Pisa, in an in vitro 3D lung model. NCI-H441 cells were seeded on collagen scaffolds, pre-formed on well inserts with 0.4 μm diameter pores and 0.33 cm^2^ area (TC-inserts, Sarstedt, Numbrecht, Germany), at a density of 40,000 cells/well in Royal Park Memorial Institute 1640 medium (RPMI) added with 10% fetal bovine serum (FBS) and 2 mM L-glutamine [complete RPMI]. Complete RPMI was added to the inferior chamber of the wells, as well. Cells in the upper chamber were incubated in a humidified atmosphere containing 5% CO_2_ for 24 h at 37 °C to reach approximately 90–100% confluence. Non-adherent cells were then removed by washing, and adherent cells were added with 100 μL of LRM-CFS diluted at 1:4, 1:6, and 1:8 in complete RPMI, with 100 μL of MRSB diluted at 1:4 in complete RPMI (CTRL) or with 100 μL of complete RPMI (neg control), and further incubated for 7 h and 24 h at 37°C, as stated above. At the end of the incubation, the wells were washed once with 200 μL warm PBS, and the collagen scaffolds were digested with 5 mg/mL collagenase for 1 h at 37°C. The cells were collected by centrifugation at 700× *g* for 5 min, and the pellets were subjected to a 3 min treatment at 37 °C with Trypsin/ethylenediaminetetraacetic acid (EDTA) to dissociate eventual cell aggregates. The pellets were washed once with PBS (700× *g*) and were resuspended in PBS. Finally, the cells were stained with 0.1 μg/mL propidium iodide (PI, Sigma Aldrich) for 5 min at room temperature, and 50,000 events were acquired ungated by using a flow cytometer (BD Accuri C6, BD Biosciences, Milan, Italy). The percentage of PI-positive dead NCI-H441 cells was calculated by computer-assisted analyses (BD Accuri C6 software version 1.0.264.21, BD Biosciences), and the percent of vitality was calculated according to the formula: 100 − [100 × (%PI pos sample − % neg control)]/(100 − % neg control).

### 4.10. Statistical Analysis

Data were analyzed using GraphPad Prism (Dotmatics, Boston, MA, USA). All the experiments were performed at least three times. Differences between mean values were evaluated by one-way or two-way analysis of variance (ANOVA), followed by the Tukey–Kramer post hoc test. A *p*-value of < 0.05 was considered significant.

## Data Availability

The data presented in this study are available on request from the corresponding author.

## Supplementary Materials

The following supporting information can be downloaded at: https://www.mdpi.com/article/10.3390/ijms25137113/s1.
