# Supplementary material for: Anti-Persisters Activity of Lacticaseibacillus rhamnosus Culture Filtrates against Pseudomonas aeruginosa in Artificial Sputum Medium
Source: Int J Mol Sci. 2024 Jun 28;25(13):7113. doi: 10.3390/ijms25137113 (PMC11241021; doi:10.3390/ijms25137113)
Supplement: Supplementary file 1 [file ijms-25-07113-s001.zip › ijms-3016342-supplementary.pdf]

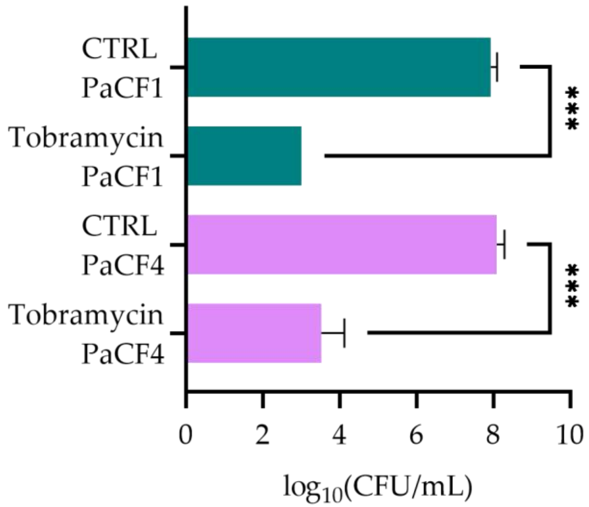

Supplementary Figure S1: Killing activity of tobramycin against *P. aeruginosa* in exponential growth phase in ASM. PaCF1 and PaCF4 grown up to exponential phase (10^8 CFU/ml) were incubated in ASM with tobramycin 128 µg/ml for 24h. The samples were plated on TSA and CFU count was performed after 24-48h. Statistical significance was evaluated by One-way ANOVA followed by the Tukey-Kramer post hoc test. ***$p<0.001$.
